# Use of antibiotics and risk of breast cancer: a population-based case–control study

**DOI:** 10.1038/sj.bjc.6602313

**Published:** 2004-12-21

**Authors:** H T Sørensen, M V Skriver, S Friis, J K McLaughlin, W J Blot, J A Baron

**Affiliations:** 1 Department of Clinical Epidemiology, Aarhus University Hospital, Vennelyst Boulevard 6, Building 260, DK-8000 Aarhus C, Denmark; 2 Institute of Cancer Epidemiology, The Danish Cancer Society, Strandboulevarden 49, 2100 Copenhagen Ø, Denmark; 3 International Epidemiology Institute, Rockville, 20850, MD and Department of Medicine, Vanderbilt University Medical Center and the Vanderbilt-Ingram Cancer Center, Nashville, 37235 TN, USA; 4 Departments of Medicine and Community and Family Medicine, Dartmouth Medical School, Hanover, 03755-3861 NH, USA

**Keywords:** breast cancer, antibiotics, risk, case–control study, prescriptions

## Abstract

We examined the use of antibiotics among 2728 women with a first diagnosis of breast cancer during 1994–2003, and 27 280 population controls in North Jutland County, Denmark, based on hospital discharge diagnoses, prescription use from 1989 to 2002, and population registry data. We found no increased relative risk of breast cancer associated with use compared with nonuse. The odds ratio for breast cancer associated with more than 10 prescriptions for antibiotics was 1.00 (95% CI 0.86 –1.15). Relative risks were similar for different classes of antibiotics. A subanalysis based on cases and controls younger than 70 years of age, with data on first birth and number of children, showed similar risk estimates even after adjustment for age at first birth and parity. In our study, use of antibiotics was not associated with an increased risk of breast cancer.

Breast cancer is the most commonly occurring cancer in women, but we have only limited knowledge about nonhormonal risk factors for this malignancy (Hankinson and Hunter, 2002). Recent case–control (Velicer *et al*, 2004) and cohort (Knekt *et al*, 2000) studies have suggested that use of antibiotics may increase the risk of breast cancer (Knekt *et al*, 2000; Velicer *et al*, 2004). A mechanism for any causal association is unknown, but it has been hypothesised that antibiotics may affect the ability of intestinal microflora to metabolise phytochemicals from edible plants into compounds that may protect against cancer (Setchell *et al*, 1981; Velicer *et al*, 2003). Since antibiotics are commonly used and breast cancer is common, any increased risk has major public health potential.

To further evaluate this association, we conducted a population-based case–control study of breast cancer in Denmark, a country with one of the highest incidences of breast cancer worldwide (National Board of Health, 2003).

## METHODS

The National Health Service in each Danish county provides health care for all residents, with free access to general practitioners and hospitals, and also refunding a variable proportion of the costs of prescribed drugs. All services are registered by use of the unique 10-digit civil registry number that is assigned to all Danish citizens shortly after birth. Its use allows valid linkage between population-based registries and the creation of a complete hospital discharge and prescription history for each individual.

### Cases with breast cancer

The Hospital Discharge Registry of North Jutland County retains key information on all patients discharged from nonpsychiatric hospitals in the county since 1977 (Andersen *et al*, 1999; Dalton *et al*, 2003; Floyd *et al*, 2003). Registry files include information on the civil registry number of the patient, dates of admission and discharge, and up to 20 discharge diagnoses (Andersen *et al*, 1999). Diagnoses are assigned exclusively by the doctor of record according to the Danish version of the International Classification of Diseases, 8th revision, and the 10th revision thereafter (Andersen *et al*, 1999).

All patients with an incident diagnosis of breast cancer (ICD8: 174.00, 174.01, 174.02, 174.08, 174.09 and ICD10: C50.0–C50.6, C50.8, C50.9) were identified in the period 1 January, 1994–31 March, 2003 (*n*=2728) using the Hospital Discharge Registry.

### Selection of controls

Controls were identified in the Civil Registration System, which has had electronic records on all changes in vital status, including change of address, date of emigration, and date of death for the entire Danish population since 1968 and 10 controls were randomly selected for each case, matched by year of birth and county of residence. A total of 10 controls were selected using incidence density sampling, that is, the controls were residents in North Jutland County, alive and at risk for a first hospital admission for breast cancer at the time the corresponding case was diagnosed, but could be subsequently diagnosed as a case. A total of 27 280 controls were included in the study.

### Data sources

The population-based Pharmaco-Epidemiological Prescription Database of North Jutland, established in 1989, was used to identify all prescriptions filled for antibiotics between 1989 and 2002 (Dalton *et al*, 2003; Floyd *et al*, 2003; Sørensen *et al*, 2004). The county is served by pharmacies equipped with computerised accounting systems through which data are sent to the Danish National Health Service and to the Prescription Database, with key information about prescriptions for refundable drugs. Thus, the database includes information on each patient's civil registry number, the type of drug prescribed according to the Anatomical Therapeutical Chemical classification system (ATC), and the date the prescription was filled. We identified all prescriptions for antibiotics (ATC codes: Penicillins: J01C, Cephalosporins: J01D A01, J01D A03, J01D A06, J01D A10, J01D A11, J01D A13, J01D A24, Macrolides: J01F, Tetracyclines: J01A, Quinolones: J01 M; Sulphonamides: J01E B02, J01E E01) among cases and controls redeemed before the date of hospitalisation of cases. These drugs are available in Denmark only by prescription. Before 1996, prescriptions to children were marked with a special code and issued to the parents, but afterwards were issued in the child's civil registry number; all prescriptions to children were excluded from the analysis. Reimbursement for tetracyclines was withdrawn in 1997. Prescriptions for postmenopausal hormone replacement therapy were also identified in the database (ATC codes: G03C, G03D, G03F, G03 H B01) and categorised as 0, 1–5, 6+prescriptions. Age at first birth and number of children were available only for women below 70 years, ascertained through linkage to the Danish Civil Registration System.

### Statistical methods

We used conditional logistic regression to estimate odds ratios (OR)s and associated 95% confidence intervals (CIs) for breast cancer according to the number of prescriptions of antibiotics and different types of antibiotics, adjusted for postmenopausal hormone replacement therapy in the overall analysis. We adjusted for age at first birth, parity, and use of postmenopausal hormone replacement therapy in cases (*n*=1917) and controls (*n*=19 170) less than 70 years of age. Wald tests were used to test for homogeneity.

## RESULTS

Table 1 provides summary data for the 2728 cases and 27 280 controls. The mean age was 62 years for both cases and controls.

Table 1 gives the ORs for breast cancer according to number of antibiotic prescriptions. Almost all risk estimates were close to 1.0. The ORs for breast cancer associated with more than 10 prescriptions for antibiotics was 1.00 (95% CI 0.86–1.15). There were no substantial differences in findings for various classes of antibiotics (penicillins, cephalosporins, macrolides, tetracyclines, quinolones, and sulphonamides) (Table 2 ). In women less than 70 years of age, the adjusted OR for breast cancer was 1.11 (95% CI 0.93–1.32) for women with more than 10 prescriptions.

Relative risks did not vary substantially between parous and nulliparous women (data not shown).

## DISCUSSION

In this large population-based case–control study, we found no increased risk of breast cancer in association with antibiotic use. Our study thus does not confirm findings from the Seattle case–control study of 2266 women with breast cancer and 7953 controls (Velicer *et al*, 2004). In that investigation, the relative risk was 1.56–2.66 for more than 10 prescriptions, but the effect was also seen in the category of 1–10 prescriptions with an OR of 1.49 (95% CI 1.28 –1.79) (Velicer *et al*, 2004). In a subanalysis, an increased risk of fatal breast cancer was seen for all antibiotic classes (Velicer *et al*, 2004). In a Finnish cohort study of women younger than 50 years of age, self-reported antibiotic use for urinary tract infections was associated with a breast cancer relative risk of 1.74 (95% CI 1.13 –2.68) (Knekt *et al*, 2000).

Several issues are relevant to the interpretation of our data. The main strengths of this study are its large size, the population-based design based on the Danish health-care system, and the use of exposure data collected before hospitalisation for breast cancer. These data sources have been extensively used in previous pharmacoepidemiological studies (Pedersen *et al*, 1999; Sørensen *et al*, 2000; Nielsen *et al*, 2001; Dalton *et al*, 2003; Floyd *et al*, 2003; Sørensen *et al*, 2004). A limitation is the shorter latency period of maximum 15 years relative to the Seattle study in which the maximum period of exposure to antibiotics was 25 years (Velicer *et al*, 2004). Also, we had no information for certain breast cancer risk factors such as alcohol consumption. However, confounding factors would have to be related to antibiotic prescriptions and inversely related to breast cancer risk, conditionally adjusted for age, which is an unlikely combination. No restrictions were imposed concerning the length of time cases or controls should have lived in North Jutland County. We obtained prescription data on cases for a mean number of days of 3478 (median: 3508) and, for controls, 3468 (median: 3502), or approximately 9.5 years.

In conclusion, the present data showed no increased breast cancer risk among antibiotic users in a population characterised by one of the highest breast cancer incidence rates worldwide (National Board of Health, 2003), although further study would be useful.

## Figures and Tables

**Table 1:** Risk of breast cancer according to number of prescriptions for antibiotics

	**Cases**		**Controls**			
**No. of prescriptions**	**No.**	**%**	**No.**	**%**	**OR**	**95% CI**
0	489	17.9	5047	18.5	1 (reference)	—
						
1–5	1327	48.6	13 078	47.9	1.02	0.91–1.14
						
6–10	467	17.1	4859	17.8	0.94	0.82–1.08
						
>10	445	16.3	4296	15.7	1.00	0.86–1.15
						
Wald test for homogeneity *P*=0.35						

**Table 2:** Risk of breast cancer according to types of antibiotics

		**Cases**		**Controls**			
**Type of antibiotic**		**No.**	**%**	**No.**	**%**	**OR**	**95% CI**
Penicillins	Overall	1974	72.4	19 589	71.8	1.00	0.91–1.10
	More than 10 prescriptions	204	7.5	2087	7.7	0.94	0.80–1.12
							
Cephalosporins^a^	Overall	1	0.0	24	0.1	0.41	0.06–3.01
	More than 10 prescriptions	0	—	6	0.0	—	—
							
Macrolides	Overall	891	32.7	8381	30.7	1.07	0.98–1.17
	More than 10 prescriptions	22	0.8	260	1.0	0.85	0.55–1.31
							
Tetracyclines	Overall	344	12.6	3202	11.7	1.05	0.93–1.19
	More than 10 prescriptions	11	0.4	73	0.3	1.44	0.76–2.73
							
Quinolones	Overall	195	7.2	1828	6.7	1.02	0.88–1.19
	More than 10 prescriptions	4	0.1	35	0.1	1.05	0.37–2.95
							
Sulphonamides	Overall	818	30.0	8468	31.0	0.92	0.84–1.00
	More than 10 prescriptions	22	0.8	285	1.0	0.68	0.44–1.06

a In Denmark, cephalosporins are almost solely used in hospitals.
